# Biomarker detection using corrected degree of domesticity in hybrid social network feature selection for improving classifier performance

**DOI:** 10.1186/s12859-023-05540-5

**Published:** 2023-10-30

**Authors:** Hatice Yağmur Zengin, Erdem Karabulut

**Affiliations:** Department of Biostatistics, Hacettepe University Faculty of Medicine, Sıhhiye, 06230 Ankara, Türkiye

**Keywords:** Social network feature selection, Dimension reduction, Classification, Biomarkers, Genomics, R genomics, 91D30, 92B15

## Abstract

**Background:**

Dimension reduction, especially feature selection, is an important step in improving classification performance for high-dimensional data. Particularly in cancer research, when reducing the number of features, i.e., genes, it is important to select the most informative features/potential biomarkers that could affect the diagnostic accuracy. Therefore, researchers continuously try to explore more efficient ways to reduce the large number of features/genes to a small but informative subset before the classification task. Hybrid methods have been extensively investigated for this purpose, and research to find the optimal approach is ongoing. Social network analysis is used as a part of a hybrid method, although there are several issues that have arisen when using social network tools, such as using a single environment for computing, constructing an adjacency matrix or computing network measures. Therefore, in our study, we apply a hybrid feature selection method consisting of several machine learning algorithms in addition to social network analysis with our proposed network metric, called the corrected degree of domesticity, in a single environment, R, to improve the support vector machine classifier’s performance. In addition, we evaluate and compare the performances of several combinations used in the different steps of the method with a simulation experiment.

**Results:**

The proposed method improves the classifier’s performance compared to using the whole feature set in all the cases we investigate. Additionally, in terms of the area under the receiver operating characteristic (ROC) curve, our approach improves classification performance compared to several approaches in the literature.

**Conclusion:**

When using the corrected degree of domesticity as a network degree centrality measure, it is important to use our correction to compare nodes/features with no connection outside of their community since it provides a more accurate ranking among the features. Due to the nature of the hybrid method, which includes social network analysis, it is necessary to investigate possible combinations to provide an optimal solution for the microarray data used in the research.

## Background

The need to analyze high-dimensional data has led to researchers combining advanced machine learning and statistical methods, especially in the genetics field. Particularly in cancer research, where data are obtained by microarrays, identifying “biomarker” genes can potentially improve diagnostic accuracy for applying individual therapeutic or preventive treatments [1, 2]. Therefore, an important part of cancer research is discovering cancer subclasses and identifying the most informative genes for each subclass. Although unsupervised learning methods are frequently used in the literature in these studies because the classes to which the individuals belong are generally not known [3], comprehensive studies have been conducted on feature selection and classification methods in the field of supervised learning [1].

In a classification task, biomarker identification can be defined as selecting the most informative subset of features to increase the classification performance of the learning algorithm. The steps of biomarker identification include feature selection, classification model construction, and validity assessment [1].

Social network analysis, a popular method that has been used for biomarker discovery in recent years, is a complex technique that is used to define the network and the structure of the entities in that network by focusing on the properties of the ties between the nodes, rather than the specific features of the entities represented by the nodes, and examines the relationships and interactions that cannot be easily observed between entities in detail. In addition, this technique provides a different way for researchers to visualize complex systems such as metabolic networks, cell-cell or protein-protein interactions, coexpression in genomics and gene regulation networks [4–7], in which both the entities and structure of the ties are taken into consideration [8, 9].

Although its success is more common due to the success in visualizing complex networks, social network analysis can be used within hybrid feature selection methods to reduce the size of high-dimensional data and identify disease-specific biomarkers [5, 6]. However, because multiple machine learning, data mining and network analysis methods can be combined, it is important to find the most effective combination specific to the problem at hand [5, 10].

The leukemia dataset, which is examined in many studies and used in the scope of this study, was first used by Golub et al. [11] who tried to differentiate acute myeloid leukemia (AML) and acute lymphocytic leukemia (ALL) classes by combining class discovery and prediction approaches. Since then, studies aiming to develop diagnostic tools that are more accurate than traditional methods have received widespread attention. In these studies, different methods have been used to obtain the optimal solution to the problem of assigning individuals to known classes. For example, to classify the leukemia dataset correctly, both traditional statistical methods, such as logistic regression [12], Fisher linear discriminant analysis [13, 14], step-wise cross-discriminant analysis [15], diagonal linear discriminant analysis [16], and partial least squares [17], and machine learning methods, such as decision-tree [18], weighted voting [19], support vector machine (SVM) [20, 21], random forest (RF) [22], deep learning [23] and Bayesian networks [24], are used.

In particular, due to the problems caused by high-dimensional and noisy data, researchers have favored machine learning methods such as nearest neighbor, SVM, RF, and artificial neural networks (ANNs), particularly hybrid approaches [25] although the definitive superiority of a single classification method has not been proven [26].

For instance, Guyon et al. [27] aimed to improve classification performance by using recursive feature elimination (RFE) with SVM as an embedded feature selection method called support vector machine-recursive feature elimination (SVM-RFE). In the study, researchers compared SVM-RFE with other methods in terms of classification performance. Additionally, there are studies in which different machine learning methods are combined with feature selection methods, such as random forest recursive feature selection (RF-RFE) [28].

Ozyer et al. [5] proposed a hybrid method called social network feature selection (SNFS), which combines social network analysis, feature selection, and clustering methods. They succeeded in decreasing the number of selected genes while increasing the classification performance of classifiers such as SVM and J48 for several open source datasets, including leukemia [11] and colon cancer [29] datasets. While social network analysis provides the opportunity to visualize complex networks, it is quite simple to create a coexpression network in genomics. In a coexpression network, the nodes represent genes, and usually, the degree of coexpression between a pair of genes describes the interaction between the two genes. Although the measures used to define the interaction between genes differ among studies [5, 30–32], similarity measures are often used for this purpose.

In this study, we aim to briefly overview several methods used in the different stages (dimension reduction, clustering, community detection) in SNFS; apply SNFS in a single environment, R [33], using two-class, open-source microarray datasets, namely, leukemia and colon cancer datasets; and compare the effects of the combinations of several methods used in the steps of SNFS on support vector machine classifier performance by a simulation study. Hence, we aim to provide optimal combinations under specific scenarios. The rest of the paper is organized as follows: section "Methods" is a brief overview of the methods combined in the steps of SNFS in addition to an introduction to our proposed network degree centrality metric, which we call the “corrected degree of domesticity”. In section "Results", the results of open-source microarray datasets and the simulation study results are presented. Finally, in sections " Discussion" and "Conclusion", we discuss and conclude our findings.

## Methods

SNFS is a hybrid feature selection method consisting of three main stages [5]. In the first step, the ranks of the genes obtained by the feature selection methods are combined, and a user-specified percentage of the genes from the combined list are selected as candidate genes. The second step is calculating the means of the gene expression levels corresponding to each class to obtain the reduced data for clustering and using repetition of the k-means clustering to calculate the number of co-occurrences of the gene pairs in the same cluster to create an adjacency matrix. In the last step, social network analysis is implemented with the weighted adjacency matrix obtained in the second step to apply a community detection algorithm. The candidate genes in each community are selected as biomarkers by combining the network-specific metrics. At the end of the third step, the validity of the method and the potential value of the genes as biomarkers can be evaluated in terms of classification performance using hold-out validation. We carried out the calculations by RStudio Version 1.4.1106 with R$-$4.0.4 installed on Windows 10 64-bit OS running on a PC with a system configuration Intel(R) Core(TM) i5-8400 CPU @ 2.80 GHz 2.81 GHz with 8.00 GB of RAM.

### Social network feature selection in R

Since the SNFS consists of multiple methods, the selection of the combinations can change the classification performance of the classifier for the same dataset. Therefore, it is important to examine the changes and compare the results to select an optimal solution.

We prepare the function to implement all the stages of the SNFS with user-specified combinations and return the results related to the classification performance of the support vector machine classifier (available at our GitHub repository). Our R function can be altered for other machine learning methods as well. We use SVM since in the literature, it is a commonly used technique when evaluating the performance of the feature selection approach for microarray data in cancer classification problems [34–37]. The parameters of the SVM with the radial basis function (RBF) kernel are optimized using the tune.svm function in R. We briefly introduce the methods used in the steps of the SNFS in the following subsections.

### Feature selection algorithms

Several dimension reduction methods have been proposed in the literature to overcome the problems that can occur during the analysis of high-dimensional data and improve the machine learning algorithm’s performance.

Dimension reduction methods are grouped into two main categories: feature selection and feature extraction. Although both approaches have their own advantages, feature selection methods are used for biomarker detection studies because the original features are important for model interpretation and information extraction since they reduce the size by removing irrelevant or redundant features while preserving the original features [10].

Feature selection methods are generally divided into four subgroups: “filters”, “wrapper methods”, “embedded methods” and “hybrid methods” [10, 38]. In the first step of the SNFS, chi-square (CS) and information gain (IG) filters, which are considered classical filters, are used [39, 40] in addition to the embedded method support vector machine recursive feature elimination (SVM-RFE) [27]. Apart from the ease of application, one of the main reasons for using these filters or embedded methods is the necessity of obtaining an objective order for the genes by which to reduce the number of genes at this stage. However, at this step, any of the sorting filters or dimension reduction techniques can be adapted to the SNFS method. Although many studies in the literature have described the benefits of the feature selection process, most researchers agree that there is no one method that can be called the best method. Therefore, problem-specific feature selection methods that implement different strategies are constantly being developed. Therefore, it is very important to evaluate and compare the performance with simulation studies.

In R, we use the FSelector package [41] to implement CS and IG filters in addition to random forest, and we implement SVM-RFE [27] with the SQRT-RFE approach [42] to reduce the computational workload. For implementation of the SVM, the e1071 package is used [43].

Then, we combine the ranked feature lists obtained from different feature selection methods and select a user-defined percentage of features as candidates.

### (Bi)clustering

Before implementing the social network analysis, it is crucial to create an adjacency matrix. Therefore, repetition of the k-means clustering method [44] is used to create the adjacency matrix needed for social network analysis. The purpose of this step is to group similar genes. However, the number of k clusters is determined by the user (k = 3, 4 or 5). In other words, there is a different subversion of the method for each number of k sets. In the literature, there is a prespecified number for which one should repeat the k-means clustering method for the number of k clusters in the SNFS [5].

In our study, the k-means clustering algorithm is run 3 times, with k cluster numbers of 3, 4 and 5. However, it is possible to increase the repetition number using the SNFS function. Using the obtained cooccurrences, the weighted adjacency matrix is calculated. In other words, the k-means clustering method is repeated multiple times, and the cooccurrences of genes are counted. In this way, a pxp square matrix is created in which the smallest value of its elements is 0 and the largest value is the “number of repetitions of the clustering method”.

In R, “kmeans” function in the stats package [33] was used with k parameters 3, 4 and 5 for each number of k clusters. The proposed function performs this step automatically.

### Social network analysis and community detection

Community detection is a process for identifying clusters in the network. Although there are several different methods in the literature, the most common methods are the Girvan-Newman; Clauset, Newman, and Moore; Pons and Latapy; Watika and Tsurumi; and Louvain methods [45]. Although only the Louvain method is used to detect communities in SNFS [5], other methods could be considered as an alternative, because the Infomap method can outperform the Louvain method [46], and the Walktrap method also performs better [47]. Therefore, in our study, other than the Louvain method, these two methods are included as alternatives.

In R, this integrated step is implemented with the igraph package [48] using the weighted adjacency matrix. Additionally, we use the R implementation of the Force Atlas 2 graph layout [49] as in Ozyer et al. [5] to construct network graphs. The cluster_louvain function is used to implement the Louvain community detection method to identify the communities in the network. However, this package also includes community detection methods such as Walktrap and Infomap, which were mentioned above. After the communities are identified, the genes can be evaluated with network-specific metrics, and biomarkers can be determined by evaluating one metric or a combination of metrics in SNFS. In other words, if a gene has a high level of interaction with members of its own community and a low level of interaction with members outside of its own community, that gene is considered to have a good ability to represent its community. Several network-specific metrics can be used, such as “coverage”, “corrected degree of domesticity”, “intracommunity unweighted degree centrality”, “out-of-community unweighted degree centrality”, “unweighted degree centrality” and “weighted degree centrality”. Although each of these metrics can be calculated, we focus on the corrected degree of domesticity ($z_{d}$) of node i, which is defined as follows: (Ozyer et al. [5]),

1 $$\begin{aligned} z_{d}(i)=z_{in}(i)/z_{out}(i) \end{aligned}$$

where $z_{in}$ represents the number of the node’s edges inside of its own community and $z_{out}$ represents the number of the node’s edges outside of its own community. As seen from Eq. 1, the corrected degree of domesticity is undefined when a node has no edges outside its own community. Therefore, nodes with no edges outside their own community cannot be compared among themselves in terms of the corrected degree of domesticity. In other words, the effect of the number of ties established by the respective node in its community cannot be evaluated. For this reason, we overcome this problem by adding a very small value, i.e., $\epsilon$ (epsilon), to both the numerator and the denominator of Eq. 1. Accordingly, the corrected degree of domesticity can be defined as follows:

2 $$\begin{aligned} z_{d}(i)=[z_{in}(i)+\epsilon ]/[z_{out}(i)+\epsilon ] \end{aligned}$$

In this study, we select biomarker genes according to the corrected degree of domesticity (Eq. 2). However, users can choose to combine several metrics. After ranking the genes according to the corrected degree of domesticity, a predefined percentage of the genes is identified as biomarkers from each community. In our study, the percentage is 10% for both the real data application and the simulation study.

**Fig. 1:**
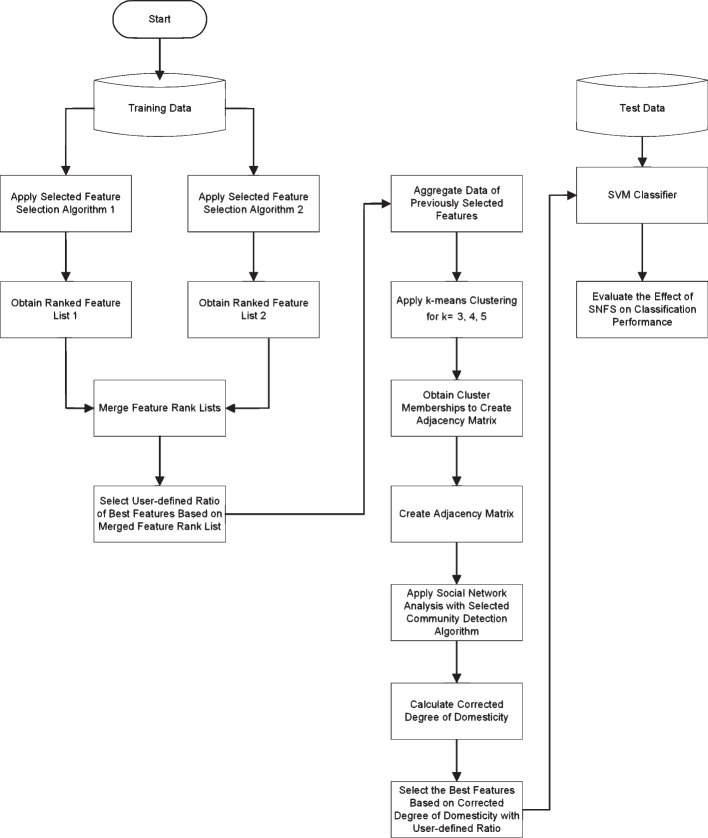
Flowchart of the SNFS using the corrected degree of domesticity

After the selection of biomarker genes, the classification performance of SVM is evaluated using the hold-out validation method for AUC, sensitivity, and specificity (Fig. 1).

#### Simulation study

In the literature, there are several approaches for data generation to evaluate the performance of hybrid methods in classification improvement. We use a strategy called the “two-group dependency structure” introduced by Guo et al. [50] due to the similarity between synthetic and real microarray gene expression. Therefore, p = 10,000 genes are generated with a total of 200 training and 600 test samples, with an equal number in each of the two classes. A total of 10,000 genes are divided into k = 100 blocks, each containing 100 genes. It is assumed that the genes in different blocks are independent of each other and that the genes in the same block are related to the covariance structure shown in Eq. 3. In the same block, $|\rho |=0.9$ (positive for 50 blocks in the training set and negative for 50 blocks). In addition, we consider $|\rho |=0.9$ to evaluate the impact of the correlation strength on classification performance. First, the expression levels of each gene are generated from the standard normal distribution, and then the expression levels are multiplied by the square root of the covariance matrix shown below. Thus, the expressions are transformed to follow a multivariate normal distribution with MVN (0, $\Sigma$). Finally, we add a constant value of 0.5 to all expression levels in the first 200 genes of the second class, as mentioned in Guo et al. [50].

3 $$\begin{aligned} \Sigma = \begin{pmatrix} 1 &{} \rho &{} \cdots &{} \rho ^{98} &{} \rho ^{99}\\ \rho &{} 1 &{} \ddots &{} \ddots &{} \rho ^{98}\\ \vdots &{} \ddots &{} \ddots &{} \ddots &{} \vdots \\ \rho ^{98} &{} \ddots &{} \ddots &{} \ddots &{} \rho \\ \rho ^{99} &{} \rho ^{98} &{} \cdots &{} \rho &{} 1 \end{pmatrix} \end{aligned}$$

We use the simdata_guo function in the sortinghat package [51] for this data generation process. However, this function does not include adding a constant value of 0.5 to prespecified gene expression levels, unlike the method described in Guo et al. [50]. Therefore, we added 0.5 to the expressions manually.

*List of abbreviations* AML, acute myeloid leukemia; ALL, acute lymphoblastic leukemia; SVM, support vector machine; RF, random forest; ANNs, artificial neural networks; RFE, recursive feature elimination; SVM-RFE, support vector machine-recursive feature elimination; RF-RFE, random forest-recursive feature elimination; SNFS, social network feature selection; CS, chi-square; IG, information gain; SQRT-RFE, square root-recursive feature elimination; SVM SQRT-RFE, support vector machine square root-recursive feature elimination; FS, feature selection; CD, community detection method used in the third step of the algorithm; CN, number of communities detected by the community detection algorithm; Acc., accuracy; Sens., sensitivity; Spe., specificity; AUC, area under the ROC curve

## Results

### Results of the open-source datasets

The leukemia dataset, which is in the R Bioconductor golubEsets package, consists of 7129 features and 72 (47 ALL, 25 AML) samples taken from patients with acute leukemia, where the training set consists of 38 samples and the test set consists of 34 samples [52]. We use the same training and test data to obtain comparable results. Using the whole feature set in R provides test set accuracy, sensitivity, specificity and AUC values of 88.2%, 95%, 78.6% and, 91.8%, respectively.

In the first step of SNFS, 1% of the genes are selected as candidates from the combined rank list obtained by feature selection. Then, in the second step, a weighted adjacency matrix is obtained by repeating the k-means clustering for k = 3, 4 and 5. After the third step of applying community detection for social network analysis, 10% of the genes (8 to 10) representing their own community according to the corrected degree of domesticity are selected as biomarker genes. The results of the leukemia test set obtained by using our own SNFS function are summarized in Table 1. Although the differences seem insignificant, combining SVM SQRT-RFE with any other filter in addition to the Infomap community detection algorithm provides slightly higher performance in terms of AUC for the leukemia dataset.

**Table 1:** Classification performances of SNFS in the leukemia test set

FS	CD	CN	Acc.	Sens.	Spe.	AUC	FN
SVM SQRT-RFE+IG	Louvain	3	0.9706	1.0000	0.9286	0.9857	9
SVM SQRT-RFE+CS		3	0.9118	1.0000	0.7857	1.0000	9
SVM SQRT-RFE+RF		3	0.8824	0.8500	0.9286	0.9393	8
IG+CS		3	0.9118	1.0000	0.7857	0.9893	8
IG+RF		3	0.9412	1.0000	0.8571	0.9536	8
CS+RF		3	0.8824	1.0000	0.7143	0.9750	9
SVM SQRT-RFE+IG	Walktrap	4	0.9706	1.0000	0.9286	0.9821	10
SVM SQRT-RFE+CS		3	0.9118	1.0000	0.7857	1.0000	9
SVM SQRT-RFE+RF		3	0.8824	0.8500	0.9286	0.9393	8
IG+CS		3	0.9118	1.0000	0.7857	0.9893	8
IG+RF		3	0.9412	1.0000	0.8571	0.9536	8
CS+RF		3	0.8824	1.0000	0.7143	0.9750	9
SVM SQRT-RFE+IG $^1$	Infomap	2	0.9412	1.0000	0.8571	0.9929	8
SVM SQRT-RFE+CS		3	0.9118	1.0000	0.7857	1.0000	9
SVM SQRT-RFE+RF		3	0.8824	0.8500	0.9286	0.9393	8
IG+CS		3	0.9118	1.0000	0.7857	0.9893	8
IG+RF		3	0.9412	1.0000	0.8571	0.9536	8
CS+RF		3	0.8824	1.0000	0.7143	0.9750	9

FS: feature selection method used in the first step of the algorithm; CD: community detection method used in the third step of the algorithm; CN: number of communities detected by the community detection algorithm; Acc.: accuracy; Sens.: sensitivity; Spe.: specificity; AUC: area under the ROC curve; FN: number of selected features

$$^{1}$$ Selected combination

For instance, one of the so-called optimal combinations in Table 1 achieves slightly balanced performance between the training and test sets with decreased feature numbers (Table 2). The calculation time is between 59.036 and 83.38 s depending on the combination used in the SNFS.

**Table 2:** Comparison of the approaches used in the leukemia dataset

	Golub et al. [11]	Guyon et al. [27]	Liu et al. [53]	Bijlani et al. [54]	Özyer et al. [5]	Our Approach
Errors (Train)	2	0	0	0	3	1
Errors (Test)	5	0	3	1	1	2
Genes$^1$	50	6–8	1	16	9	8

$^1$Number of genes (biomarkers) used for classification with SVM

**Table 3:** Classification performances of SNFS in colon cancer test set

FS	CD	CN	Acc.	Sens.	Spe.	AUC
SVM SQRT-RFE+IG	Louvain	3	0.8947	0.8571	0.9167	0.9167
SVM SQRT-RFE+CS		3	0.8947	0.8571	0.9167	0.9167
SVM SQRT-RFE+RF		3	0.8421	0.8571	0.8333	0.8214
IG+CS		3	0.6316	0.4286	0.75	0.8214
IG+RF		3	0.8421	0.7143	0.9167	0.9643
CS+RF		3	0.8421	0.7143	0.9167	0.9405
SVM SQRT-RFE+IG	Walktrap	3	0.8947	0.8571	0.9167	0.8929
SVM SQRT-RFE+CS		3	0.8947	0.8571	0.9167	0.8929
SVM SQRT-RFE+RF		3	0.8421	0.8571	0.8333	0.8214
IG+CS		3	0.6316	0.4286	0.75	0.8214
IG+RF		4	0.8421	0.7143	0.9167	0.9524
CS+RF		4	0.8421	0.7143	0.9167	0.9643
SVM SQRT-RFE+IG	Infomap	3	0.8947	0.8571	0.9167	0.8929
SVM SQRT-RFE+CS		3	0.8947	0.8571	0.9167	0.8929
SVM SQRT-RFE+RF		3	0.8421	0.8571	0.8333	0.8214
IG+CS		3	0.6316	0.4286	0.75	0.8214
IG+RF		3	0.8421	0.7143	0.9167	0.9643
CS+RF		3	0.8421	0.7143	0.9167	0.9405

FS: feature selection method used in the first step of the algorithm; CD: community detection method used in the third step of the algorithm; CN: number of communities detected by the community detection algorithm; Acc.: accuracy; Sens.: sensitivity; Spe.: specificity; AUC: area under the ROC curve

In addition, we apply SNFS to the colon cancer dataset [29, 55], which includes expression levels of 2,000 genes and 62 samples (40 samples from tumor tissue, 22 samples from normal tissue) from colon cancer patients in the R colonCA package [55]. We use our R function to split the data into test and training sets with a user-specified ratio considering prevalence. For this dataset, we split the data into 70% and 30% for the training and test sets, respectively, corresponding to 43 samples in the training set and 19 samples in the test set. Before the SNFS, we use a preprocessing (standardization) procedure explained in Alon et al. [29].

In this study, 10% of the genes are selected from the merged feature rank list obtained from the combinations of feature selection methods. Then, k-means clustering is applied for k=3, 4 and 5 as described above for calculating the weighted adjacency matrix. After community detection in social network analysis, the top 5% of genes (between 11 and 12) that can represent each of their communities according to their corrected degree of domesticity are selected. The results obtained from the SVM in the test set using the selected genes are represented in Table 3. Using the whole feature set only provides an accuracy, sensitivity, specificity, and area under the ROC curve of 83.3%, 85.7%, 80%, and 82.1% respectively. On the other hand, although there are small differences in selecting different community detection algorithms, selecting biomarker genes with SNFS, especially combining IG and RF, usually provides higher AUC values. The calculation time was between 32.332 and 82.997 s depending on the combination used in the SNFS.

### Results of the simulation study

After the data generation process introduced by Guo et al. [50], the SNFS method is applied with hold-out validation. To obtain approximately 50 informative genes out of 10,000 in the dimension reduction of SNFS, 5% of the genes are selected, and after community detection, 10% of the genes are considered the best biomarker genes according to the corrected degree of domesticity. The results of the classification performance of SVM with those biomarker genes are shown in Table 4.

**Table 4:** Results of the simulated data with rho = 0.60

FS	CD	Acc.	Sens.	Spe.	AUC
SVM SQRT-RFE+CS	Louvain	0.771	0.810	0.732	0.847
SVM SQRT-RFE+IG		0.769	0.828	0.710	0.845
SVM SQRT-RFE+RF		0.821	0.872	0.770	0.907
IG+CS		0.781	0.798	0.764	0.863
IG+RF		0.687	0.756	0.618	0.771
CS+RF		0.689	0.852	0.526	0.789
SVM SQRT-RFE+CS	Walktrap	0.740	0.676	0.804	0.811
SVM SQRT-RFE+IG		0.769	0.828	0.710	0.845
SVM SQRT-RFE+RF		0.821	0.872	0.770	0.907
IG+CS		0.737	0.682	0.792	0.829
IG+RF		0.687	0.756	0.618	0.771
CS+RF		0.689	0.852	0.526	0.789
SVM SQRT-RFE+CS	Infomap	0.771	0.810	0.732	0.847
SVM SQRT-RFE+IG		0.769	0.828	0.710	0.845
SVM SQRT-RFE+RF		0.821	0.872	0.770	0.907
IG+CS		0.781	0.798	0.764	0.863
IG+RF		0.687	0.756	0.618	0.771
CS+RF		0.689	0.852	0.526	0.789

FS: feature selection method used in the first step of the algorithm; CD: community detection method used in the third step of the algorithm; Acc.: accuracy; Sens: sensitivity; Spe.: specificity; AUC: area under the ROC curve

Although all the combinations provide acceptable results, the SVM-SQRT RFE with RF combination using any of the community detection algorithms provides the best result when SNFS is applied. However, combining the Infomap and Louvain community detection algorithms provides similar or higher classification performances compared to Walktrap in any of the scenarios using 51 to 52 genes as the biomarkers.

In addition, the classification performances of the SVM obtained by selecting between 51 and 52 genes by the SNFS method are better than using the whole feature set for both correlation levels. When the correlation between genes in the same block is $|\rho |$ = 0.60, the highest accuracy, sensitivity, specificity, and area under the ROC curve obtained from SVM in the test set are 81.5%, 80.2%, 82.8% and, 90.2%, respectively. However, the same values were 82.1%, 87.2%, 77% and, 90.7% when using SNFS, with only 51 genes out of 10,000. We obtain the results for all the combinations of the SNFS in 7190.179 s when $|\rho |$ = 0.60 for 1 iteration.

**Table 5:** Results of the simulated data with rho = 0.90

FS	CD	Acc.	Sens.	Spe.	AUC
SVM SQRT-RFE+CS	Louvain	0.675	0.604	0.746	0.760
SVM SQRT-RFE+IG		0.702	0.644	0.760	0.761
SVM SQRT-RFE+RF		0.629	0.498	0.760	0.689
IG+CS		0.683	0.636	0.730	0.733
IG+RF		0.632	0.606	0.658	0.687
CS+RF		0.632	0.606	0.658	0.688
SVM SQRT-RFE+CS	Walktrap	0.717	0.664	0.770	0.775
SVM SQRT-RFE+IG		0.702	0.644	0.760	0.761
SVM SQRT-RFE+RF		0.629	0.498	0.760	0.689
IG+CS		0.679	0.638	0.720	0.732
IG+RF		0.632	0.606	0.658	0.687
CS+RF		0.632	0.606	0.658	0.688
SVM SQRT-RFE+CS	Infomap	0.717	0.664	0.770	0.775
SVM SQRT-RFE+IG		0.702	0.644	0.760	0.761
SVM SQRT-RFE+RF		0.629	0.498	0.760	0.689
IG+CS		0.677	0.636	0.718	0.732
IG+RF		0.632	0.606	0.658	0.687
CS+RF		0.632	0.606	0.658	0.688

FS: feature selection method used in the first step of the algorithm; CD: community detection method used in the third step of the algorithm; Acc.: accuracy; Sens.: sensitivity; Spe.: specificity; AUC: area under the ROC curve

When the correlation between genes in the same block is increased to $|\rho |$ = 0.90, the highest accuracy, sensitivity, specificity, and area under the ROC curve obtained from SVM are 67.4%, 65.6%, 69.2% and, 72.5%, respectively. On the other hand, SNFS provides 71.7%, 66.4%, 77% and, 77.5% with 51 genes only (Table 5). We obtain the results for all the combinations of the SNFS in 4811.601 s when $|\rho |$ = 0.90 for 1 iteration. Accordingly, the SNFS method is a preferred method in terms of dimension reduction, especially when the correlation between genes in the same block is higher. In addition, when the correlation level within blocks is lower, classification performance tends to be higher. The increasing correlation within the block causes a decrease in classification performance in general.

## Discussion

In recent years, the use of social network analysis in the field of medicine and genetics to identify disease-specific biomarkers that will increase classification performance by reducing the size of high-dimensional data has become popular.

However, since there are many different combinations of multiple algorithms within the scope of hybrid methods, the importance of determining the optimal combination has emerged [10].

In this study, in addition to applying the hybrid method SNFS for biomarker detection using microarray data, several combinations of possible methods that can be used within the scope of SNFS are evaluated. In addition, the effects of these different combinations on the open-source microarray datasets used in the study are investigated using R software. SNFS provides an increase in the classification performance of the SVM classifier for both leukemia and colon cancer datasets compared to using the whole feature set. In addition, it provides better or similar performance compared to other studies [5, 11, 50].

**Table 6:** SNFS using CDD versus several approaches using SVM on leukemia and colon cancer datasets

Reference	CS	Dataset	FS	Time	FN	Acc.	AUC
Our approach	1	Leukemia	SNFS with CDD	71.22 (59.04-83.38)$^1$	8-10	88.24-97.06$^2$	93.93-100$^2$
Our approach	1	Colon cancer	SNFS with CDD	37.23 (32.33-83)$^1$	11-12	63.16-89.47$^2$	82.14-96.43$^2$
Ozyer et al. [5]	2	Leukemia	SNFS	NM	9	97.06	NM
Ozyer et al. [5]	2	Colon cancer	SNFS	NM	9	100	NM
Ding et al. [56]	3	Leukemia	OELM-RFE	NM	4	84.38	NM
Khaire and Dhanalakshmi [57]	NM	Leukemia	IWOA	NM	NM	81	92
Khaire and Dhanalakshmi [57]	NM	Colon cancer	IWOA	NM	NM	83	84
Kundu et al. [58]	4	Leukemia	AWOA	41.53	30	100	100
Kundu et al. [58]	4	Colon cancer	AWOA	10.37	21	100	Approx. 100
Vatankhah and Momenzadeh [59]	5	Leukemia	Self-regularized LASSO	14.97	1	NM	100

CDD: corrected degree of domesticity; CS: computational resources; FS: feature selection method; time: execution time in seconds; FN: number of selected features; Acc.: accuracy; AUC: area under the ROC curve; NM: Not mentioned; Approx.: approximately

CS: 1: Intel(R) Core(TM) i5-8400 CPU @ 2.80 GHz 8.00 GB of RAM; 2: Intel(R) Core(TM) i5 CPU @ 3.10 GHz with 4.00 GB of RAM; 3: I7-7700HQ CPU @ 2.8 GHz with 8.00 GB of RAM; 4: Intel(R) Core(TM) i5 with 8.00 GB of RAM; 5: Intel(R) Core(TM) i7-10750 H CPU @ 2.60 GHz with 16.00 GB of RAM

$^1$Median (minimum-maximum) values for execution time of the possible combinations in SNFS

$^2$Minimum-maximum values of the performance measure obtained from the possible combinations in SNFS

For instance, in the leukemia dataset, we obtain more balanced classification results in terms of training and test set differences and better results in the training set than in Ozyer et al. [5]. In addition, compared to some studies in the literature, better [2, 50] or similar [11] classification performance is achieved with a smaller number of genes.

In addition, when we compare our results to those of some recent studies [56–59], our approach provides similar or greater classification performance in terms of AUC in a short execution time along with the small number of selected features. Furthermore, when we consider all the possible combinations of SNFS and select the optimal combination, we obtain similar or higher accuracy compared to several other methods (Table 6).

Therefore, SNFS can be considered a successful hybrid feature selection method for these specific datasets. However, choosing different combinations in the steps of the SNFS produces slightly different results. Since embedded methods require a longer computational time, filter combinations that have a positive effect on classification performance, together with fast computational time, could be used. Similarly, as seen from the simulation study, the methods that are used in this study generally provide acceptable classification performances for the SVM classifier. Almost all feature selection and community detection method combinations result in similar classification performances in SVM. However, Infomap and Walktrap community detection methods tend to produce similar results. At the same time, the classification performance with a higher number of genes can be achieved with far fewer genes by SNFS. In the study by Guo et al. [50], 200 samples that they produced in a similar method as that of our study obtained 167 to 282 genes with the methods they proposed and reached accuracies of 89.5% and 87.5%, respectively, while 51–52 genes selected with SNFS in our study had accuracies between 99.5% to 99.9% with SVM in the training set. Although this success in the training set was noteworthy, the same classification performance is not achieved in the test set. In the work by Guo et al. [50] 90.4% to 89.2% accuracy was achieved by selecting 167 to 282 genes in the 1000 sample test set. However, when using 51 to 52 genes selected with SNFS in the 1000 sample test set in this study, accuracy can only be increased up to 82.1%. However, this result, caused by the number of features selected, is significantly lower than those in the literature.

In addition, the effect of correlation on the classification performance is observed by changing the correlation structure between the genes, and with an increase in the correlation for genes in the same block, a decrease in the classification performance obtained from the SVM is observed. Accordingly, the correlation structure between genes directly affects the classification performance, and the positive effect of dimension reduction can decrease because of the increase in correlation between genes in the same block. We observed the effect of changing one of the SNFS parameters (feature selection method combinations, percentage of genes to be selected, whether or not the clustering step is skipped, community detection methods, percentage of genes that can represent the community, network-specific metrics, etc.) by using our SNFS function. However, the effect of changes in all these parameters on the classification performance achieved with SVM can also be compared.

We advise that in the stages of hybrid feature selection methods, since there are many different combinations of multiple machine learning, data mining and/or social network analysis methods, it is necessary to evaluate all possible combinations to combine these methods appropriately and find the most suitable solution specific to the problem.

The SNFS method is a hybrid feature selection method using social network analysis and has the potential to provide different classification performances for the same dataset due to the possibility of using different algorithms and methods in multiple steps of the method. Therefore, functions have been developed so that users can apply the SNFS method on the same platform and try possible combinations of methods themselves.

The SNFS method, which is evaluated within the scope of the two-class classification problem discussed in our study, can also be adapted through the use of feature selection methods suitable for multiclass problems.

## Conclusion

In conclusion, our proposed network degree centrality measure, called the corrected degree of domesticity, can address the situation when genes have different inner community connections and no connections outside their community and makes it possible to compare and correctly order the genes in each community. As a hybrid feature selection method, SNFS can improve the classification performance of state-of-the-art models such as SVM, while users can use a single environment, R, with our R functions. SNFS produces slightly different classification performances since this method has several steps that make it possible to implement different algorithm combinations. Therefore, our functions change accordingly and provide a way to compare different combinations that the user can select. Additionally, the correlation structure of the high-dimensional dataset affects the overall performance. Hence, researchers should evaluate the correlation structure before applying the method. In future studies, we aim to investigate several combinations of feature selection algorithms in SNFS that we did not include in our current work. Investigating the effect of increasing the number of iterations in the biclustering step is another crucial research question we aim to investigate. We recommend that researchers evaluate different aspects of the SNFS method and search for an optimal combination. Furthermore, we aim to increase the efficiency of our R functions to decrease the computational time. Additionally, and most importantly, most of the studies in the literature have different preprocessing procedures, selection of classifiers, validation strategies or computational resources when evaluating the effects of the proposed feature selection algorithms although the same microarray datasets are used. In future work, we aim to conduct a comprehensive simulation study in one environment to compare results more objectively along with real data applications.

## Data Availability

The R functions developed for the current study are available in the GitHub repository (https://github.com/ygmrzngn/snfs). The datasets used during the current study include open-source datasets that are available in the R environment. The datasets generated during the current study are available from the corresponding author upon reasonable request.
